# Litter-Level Agreement of RT-qPCR Results for Influenza A Virus Between Oropharyngeal Swab, Udder Wipe, and Nasal Wipe Samples Collected in Chilean Swine Breeding Herds

**DOI:** 10.3390/ani16142261

**Published:** 2026-07-21

**Authors:** Juan Manuel Sanhueza, Nicolas Rodríguez, Daniela Tapia-Escárate, Christopher Hamilton-West, Pedro Jiménez-Bluhm, Jaime Figueroa, Macarena Cortez, Javiera Jofre, Ignacio Lopez, Rembert Silva, Cesar Corzo, Carles Vilalta, Montserrat Torremorell

**Affiliations:** 1 Departamento de Ciencias Veterinarias y Salud Pública, Facultad de Recursos Naturales, Universidad Católica de Temuco, Temuco 4780000, Chile; dtapia@uct.cl (D.T.-E.); jjofre2020@alu.uct.cl (J.J.); ilopez2022@alu.uct.cl (I.L.); rembert.silva@uct.cl (R.S.); 2 Departamento de Medicina Preventiva Animal, Facultad de Ciencias Veterinarias y Pecuarias (FAVET), Universidad de Chile, Santiago 8820000, Chile; nicolas.rodriguez.c@ug.uchile.cl (N.R.); christopher.hamilton@veterinaria.uchile.cl (C.H.-W.); 3 Escuela de Medicina Veterinaria, Facultad de Medicina, Facultad de Ciencias Biológicas y Facultad de Agronomía y Sistemas Naturales, Pontificia Universidad Católica de Chile, Santiago 7820436, Chile; pedro.jimenez@uc.cl; 4 SENTINET, Santiago 7820436, Chile; 5 Agrícola AASA LTDA, Santiago 8470472, Chile; jaime.figueroa@aasa.cl; 6 Independent Researcher, Rancagua 2820000, Chile; 7 Veterinary Population Medicine Department, University of Minnesota, Saint Paul, MN 55108, USA; corzo@umn.edu (C.C.); torr0033@umn.edu (M.T.); 8 Unitat Mixta d’Investigacio IRTA-UAB en Sanitat Animal, Centre de Recerca en Sanitat Animal (CReSA), 08193 Barcelona, Spain; carles.vilalta@irta.cat; 9 Programa de Sanitat Animal, Centre de Recerca en Sanitat Animal (IRTA-CReSA), Institut de Recerca i Tecnologia Agroalimentaries (IRTA), 08193 Barcelona, Spain

**Keywords:** influenza A, breeding herd, swine, monitoring, litter, udder wipes, nasal wipes, sensitivity

## Abstract

This study evaluated the use of udder wipes and aggregated nasal wipes as group sampling methods to detect influenza A virus (IAV) in swine breeding herds. Five breeding herds were selected and sampled from 2022 to 2024. At each sampling, six pigs in five due-to-wean litters were selected and sampled using oropharyngeal swabs (one per pig) and nasal wipes (one nasal wipe for three pigs). Additionally, an udder wipe sample was collected for each litter. Overall, 26.1% (89/341) of the oropharyngeal samples were IAV RT-qPCR positive. At the litter level, 43.9% (25/57) had at least one positive oropharyngeal swab, 33.3% (19/57) had a positive udder wipe, and 26.7% (12/45) had at least one positive nasal wipe. When compared to the oropharyngeal litter-level classification, the sensitivity and specificity of udder wipes were estimated at 68.0% (46.5–85.1%) and 93.8% (79.2–99.2%), respectively. For nasal wipes, the estimated values were 75.0% (47.6–92.7%) and 100.0% (88.1–100.0%), respectively. Udder wipes and nasal wipes can be used for IAV monitoring in breeding herds. However, false-negative results may be of concern at the litter level. These estimates can be used to implement a sampling strategy that maximizes the room- and herd-level sensitivity.

## Simple Summary

When monitoring the presence of influenza A virus (IAV) in swine populations, the use of group sampling methods such as udder wipes or oral fluids is common. However, the sensitivity of these methods to detect the virus should be evaluated to properly interpret test results from group samples. In this study, we sampled pigs from five breeding herds using oropharyngeal swabs, nasal wipes, and udder wipes and compared their litter-level RT-qPCR results to quantify the sensitivity and specificity of udder wipes and nasal wipes compared to the oropharyngeal sample results. While sample collection with udder wipes and nasal wipes is straightforward, their sensitivity estimates suggest that false-negative results can occur relatively frequently at the litter level. However, if the objective of the IAV monitoring is to detect its presence at the room or farm level, then these estimates can be useful to determine the sample size needed to achieve the desired sensitivity at the room or farm level to detect IAV.

## 1. Introduction

Influenza is a widespread global disease often caused by the influenza A virus (IAV) [1,2]. The disease is prevalent in commercial swine populations at the herd and animal levels [3,4,5]. Influenza in pigs is often associated with low mortality risk increase [6] and reduced weight gain in growing pigs [7]. IAV infection usually resolves within seven days after the onset of clinical signs [8]. However, secondary bacterial infections can complicate the clinical outcome of influenza in pigs [8,9], leading to an increased need for antimicrobial use in swine herds [10], as respiratory infections are a main driver for antimicrobial use in pig farms [11,12].

IAV is zoonotic, and people who work with animals or their environment are occupationally at risk of exposure and infection. For example, evidence of exposure has been observed in swine workers at a higher percentage than in the general population [13], and workers exposed to IAV-infected swine had higher odds of testing RT-qPCR positive at the end of the workday than at the beginning [14]. There is an interspecies barrier for IAV infections that is given by the receptors that the virus binds to infect a cell. Avian influenza viruses tend to bind to glycoconjugates with terminal alpha 2,3 linked sialic acid, while human and swine influenza viruses tend to bind to glycoconjugates with an alpha 2,6 linked sialic acid [15,16,17]. Nonetheless, both types of terminal alpha receptors are present in the trachea of pigs, making them susceptible to infection with avian, swine, and human-adapted IAV strains [16]. Coinfection of pigs with avian, swine, and human-adapted viruses may result in viral reassortment and the emergence of new influenza viruses of pandemic potential, as was the case for the 2009 H1N1 pandemic (H1N1 pdm09) [15,18]. Consequently, establishing monitoring and surveillance systems for IAV in pigs is critical to understanding viral transmission, disease occurrence, and early detection of new viruses with pandemic potential. In vivo IAV RNA detection in pigs is usually done through RT-qPCR testing of saliva or nasal secretions. From this, a range of sampling methods has been proposed to detect IAV RNA in swine populations. Sampling methods can be broadly categorized into individual (e.g., nasal swabs, oropharyngeal swabs), group (e.g., udder wipes, family oral fluids, oral fluids), and environmental sampling (e.g., surface wipes, particle deposition, air sampling) [19,20,21]. Group samples and environmental samples are easier to collect and, therefore, require less labor and time to sample a population of pigs than individual sampling. However, the ability to detect IAV in the population when it is truly present can be affected negatively when using a group or environmental sampling method. For example, when compared with pig nasal wipes, Moraes, Gauger, Osemeke, Machado, Cezar, Paiva, Mil-Homens, Almeida, Ramirez, Silva and Linhares [21] observed that the estimated sensitivity of family oral fluids, udder wipes, and sow nasal wipes at the litter level was 87% (95% CI 72–96%), 74% (95% CI 57–87%), and 42% (95% CI 26–59%), respectively, in one breeding herd in the Midwestern US.

In Chile, although AIV occurrence in commercial swine herds has not been formally assessed, Mena et al. [22] reported that 42.8% of farms and 34.4% of lung samples submitted to a laboratory prior to 2009 were positive for IAV by immunohistochemistry. Despite its apparent frequent presence in pig farms in the country, little is known about the performance of sampling methods commonly used to detect the virus in these farms. Therefore, this study aimed to evaluate the use of oropharyngeal swabs, udder wipes, and nasal wipes to detect influenza A virus in breeding herds in Chile.

## 2. Materials and Methods

Five commercial sow breeding herds (A, B, C, D, and E) from two companies located in Chile were purposely selected for inclusion in the study. Breeding herds had an inventory of 2600, 2000, 1700, 2000, and 1500 sows in farms A to E, respectively. IAV whole-herd vaccination with a custom-made vaccine was implemented in all farms from 2022 to 2023. In 2024, a commercial vaccine was used in the herds to prevent IAV infection. Herd A underwent a *Mycoplasma hyopneumoniae* elimination program in November 2022. The herd remained closed to replacement animal entry for 10 months. Farms A and D were sampled three times each in June 2022, May 2023, and May 2024. Farms B and C were sampled twice in June 2022 and June 2023. Farm E was sampled once in June 2023. At each sampling event, oropharyngeal fluids were collected individually using flocked swabs from thirty due-to-wean pigs from five litters (i.e., six pigs per litter). Each swab was inserted into the pig’s mouth and rotated from the side to the back portion of the mouth. In addition, the snout surface of the same six selected pigs was sampled using a 5 × 5 cm sterile gauze (nasal wipe) moistened with 10 mL of phosphate-Buffered Saline (PBS), ChemiX^®^, Farmalatina, Santiago, Chile. The same gauze was used to sample three pigs within each litter, resulting in two aggregated nasal wipe samples per litter in a subset of 45 litters. Finally, the skin surface of the mammary line of each sow was sampled using a 5 × 5 cm sterile gauze (udder wipe) moistened with 10 mL of PBS. To avoid cross-contamination of samples, a new pair of gloves was used for each sampled litter and across sampling methods (i.e., oropharyngeal sampling, nasal wipe sampling, udder wipe sampling) within a litter. All samples were transported chilled with ice packs on the same day of collection to the Veterinary Epidemiology Laboratory of the Universidad Católica de Temuco for processing and RT-qPCR testing. For this, the MagMAX^TM^ Viral/Pathogen Nucleic Acid Isolation Kit, Applied Biosystems, Foster City, CA, USA was used to extract IAV RNA. Samples with a cycle threshold (Ct) value <38 were considered positive for IAV.

### 2.1. RT-qPCR Positive Proportion Estimation

The proportion of RT-qPCR-positive pigs based on oropharyngeal swab results was described. In addition, the proportion of RT-qPCR-positive litters based on oropharyngeal swab, nasal wipe, or udder wipe classification was described. To estimate the proportion and 95% confidence interval of RT-qPCR-positive pigs for IAV using oropharyngeal swabs, an intercept-only generalized estimating equations (GEE) model with a binomial link function and exchangeable correlation structure was built [23,24,25]. The association between oropharyngeal swab RT-qPCR status and year of sampling and herd of origin was evaluated using a GEE model with a binomial link. The litter ID was added to each model as a cluster variable. Estimated marginal means and 95% confidence intervals (95% CI) were obtained using the emmeans R package, version 1.11.2 [26].

### 2.2. Ct-Value Dilution Effect

The Ct-value of oropharyngeal swabs was compared with the Ct-value of nasal swabs and udder wipes to quantify the Ct-value change among sampling methods within the same litter. For this, a generalized linear mixed model was built where the Ct-value was the dependent variable, and the sampling method was included in the model as an independent variable. The litter ID was included in the model as a random variable.

### 2.3. Litter Level Influenza A Virus Status Classification

Litters were classified as RT-qPCR positive or negative according to the sampling method used. A litter was classified as positive if at least one oropharyngeal swab was positive. One positive nasal wipe result was used to classify the litter as positive for IAV. Finally, a litter was also considered positive when the udder wipe was RT-qPCR positive.

### 2.4. Litter Level Agreement of Influenza A Virus Status Classification

Two-by-two contingency tables were built to evaluate the agreement between litter-level IAV status based on individual oropharyngeal swab RT-qPCR results and litter-level IAV status based on nasal wipe RT-qPCR results or udder wipe RT-qPCR results. Litter level agreement of IAV status classification between sampling strategies was assessed by estimating the total crude agreement and Kappa statistics (estimation of agreement beyond chance). Kappa values of 0.0, 0.01–0.2, 0.21–0.4, 0.41–0.6, 0.61–0.8, and 0.81–1.0 were considered as poor, slight, fair, moderate, substantial, and almost perfect agreement, respectively [27]. The McNemar test was used to statistically compare the litter-level classification based on the different sampling strategies. Additionally, sensitivity, specificity, positive predictive value, and negative predictive value of nasal wipes and udder wipes to detect IAV at the litter level were estimated considering the litter IAV status classification based on oropharyngeal swab results as the gold standard.

### 2.5. Statistical Software

R version 4.5.1 was used for data analysis and visualizations [28].

## 3. Results

**Figure 1:**
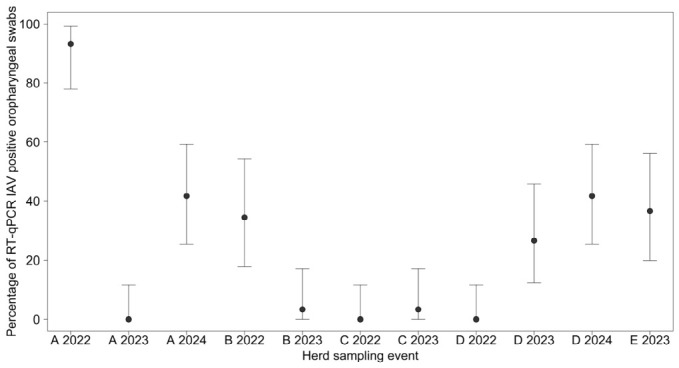
Influenza A virus RT-qPCR positive percentage and 95% confidence interval of individual oropharyngeal swabs in the five breeding herds assessed from 2022 to 2024.

A total of 341 pigs distributed in 57 litters in five breeding herds were sampled using oropharyngeal swabs. Overall, 89/341 oropharyngeal samples were RT-qPCR positive. RT-qPCR positive percentage ranged from 0/30 (0%) to 28/30 (93.3%) among the breeding herds over the years (Figure 1). The percentage of RT-qPCR-positive pigs in breeding herd A decreased from 95.6% in 2022 to 2.2% in 2023. Among the 57 litters sampled, 32 (56.1%, 95% CI 42.4–69.3%) had no RT-qPCR-positive oropharyngeal swabs in the six pigs assessed within each litter. In the 25 litters that had at least one RT-qPCR-positive oropharyngeal swab, the median within-litter positive percentage was 66.7% (1st Q 16.7–3rd Q 83.3%). Overall, the mean within-litter prevalence after adjusting for the effects of year, herd, and litter was 20.6% (95% CI 11.7–33.6%). Table 1 shows the results of the multivariable GEE model. After accounting for the effects of herd and litter, the odds of IAV RT-qPCR positivity were significantly lower in the year 2023 compared to those observed in 2022 (OR = 0.16). In addition, herd C showed lower odds of RT-qPCR positivity than herd A, after accounting for the effects of year and litter of origin.

The overall litter-level RT-qPCR positive percentage in the 57 litters evaluated was 43.9% (95% CI 30.7–57.6%) when IAV presence was assessed using oropharyngeal swabs (25/57). However, the litter-level RT-qPCR positive percentage was 33.3% (95% CI 21.4–47.1%) when assessed using udder wipes (19/57). Also, 26.7% (95% CI 14.6–41.9%) of litters had a positive RT-qPCR nasal wipe sample (12/45).

**Figure 2:**
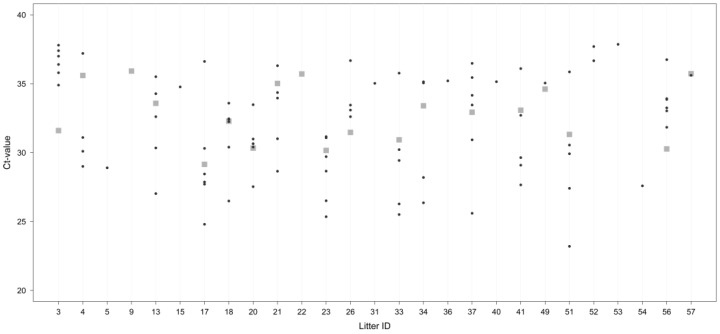
RT-qPCR Ct-values of udder wipe samples (squares) and oropharyngeal swabs of individual pigs (dots) within a litter.

The median Ct-value of the 89 positive oropharyngeal swabs was 32.5 (1st Q = 29.1–3rd Q = 35.1). At the litter level, Ct-values of udder wipes ranged from 29.2 to 35.9 and had a median value of 32.9 (1st Q = 31.1–3rd Q = 34.8). Aggregated nasal wipe positive samples had a median Ct-value of 31.8 (1st Q = 29.9–3rd Q = 34.5). Figure 2 shows the observed Ct-values of oropharyngeal swabs and udder wipes in 27 litters that were positive for either sampling method. Among the 17 litters with positive RT-qPCR results in both oropharyngeal and udder wipe samples, the mean difference between Ct-values was 0.58 (95% CI −1.0–2.2), indicating that udder wipes were, on average, less than one Ct higher than the Ct observed in the individual oropharyngeal swabs. This difference was not statistically significant (*p* = 0.48).

**Figure 3:**
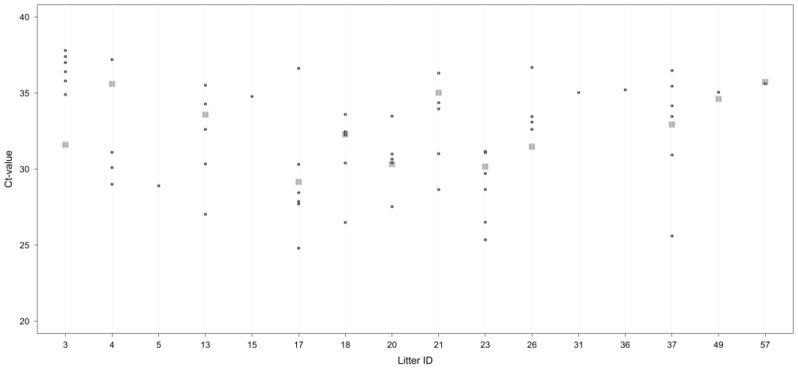
RT-qPCR Ct-values of nasal wipe samples (squares) and oropharyngeal swabs of individual pigs within a litter (dots).

There were 12 litters in which positive RT-qPCR results were obtained in both oropharyngeal swabs and nasal wipes. Nasal wipe Ct-values were on average half a Ct higher than those observed in oropharyngeal swabs (mean = 0.46; 95% CI −1.8–2.7). The difference was not statistically significant (*p* = 0.69). Figure 3 shows the observed Ct-values of oropharyngeal swabs and nasal wipes in litters that have either an RT-qPCR positive oropharyngeal swab or a positive nasal wipe among the 45 litters that were sampled using both methods.

Table 2 shows the litter level agreement between IAV classification based on udder wipes and oropharyngeal swabs. A substantial agreement between the sampling strategies was observed (kappa = 0.63). No significant statistical difference was observed in the IAV status classification by the two sampling methods (McNemar *p*-value = 0.11). The sensitivity of udder wipes to correctly classify a litter as positive when at least one out of the six pigs sampled in a litter had an RT-qPCR-positive oropharyngeal swab was 68.0% (95% CI 46.5–85.1%).

Nasal wipes were able to detect 75% (95% CI 47.6–92.7%) of litters that had at least one out of six pigs positive for IAV in oropharyngeal swabs. Litter classification agreement between using nasal wipes and oropharyngeal swabs was substantial with an estimated Kappa value of 0.8 (95% CI 0.51–1.00). Table 3 shows the litter-level agreement between IAV classification using oropharyngeal swabs or nasal swabs.

## 4. Discussion

This study investigated the agreement between three sampling strategies to classify the IAV status of a given due-to-wean litter of pigs. A substantial agreement was observed at the litter level between oropharyngeal swab RT-qPCR results and udder wipe RT-qPCR results (Kappa = 0.63). A similar agreement (kappa = 0.75) between udder wipes and oropharyngeal swabs was observed by Garrido-Mantilla et al. [19] in US breeding herds. Udder wipe RT-qPCR results were also observed to correlate (Kappa = 0.74) with nasal swab RT-qPCR results in Germany [29]. Udder skin wipes most likely gather pre-weaned pigs’ oral and nasal secretions that are left in the mammary line of lactating sows [19]. Nonetheless, it cannot be ruled out that the source of the RNA present in the sample is also environmental (i.e., originating from another litter). In our data, two litters out of the 57 had an RT-qPCR-positive udder wipe, while oropharyngeal samples were RT-qPCR-negative. However, since we sampled six pigs from the litter, it is unknown whether there was an IAV-infected pig within the litter that was not sampled. In any case, this happened in 3.5% of the sampled litters; therefore, it was not a frequent event in our data.

Despite the substantial agreement observed between sample types, it is important to note that false-negative results at the litter level can occur when using udder wipes to detect IAV. The estimated litter sensitivity of udder wipes was 68.0% (46.5–85.1%) when compared to oropharyngeal swabs. Moraes et al. [21] compared IAV detection in litters using udder wipes and nasal wipes. The authors estimated the sensitivity of udder wipes at 74% (57–87%) and observed substantial agreement between the two sampling methods (Kappa = 0.65). Although the method of sampling used as a comparison (i.e., nasal wipes) was different from the one used in our study (i.e., oropharyngeal swabs), the litter level sensitivity estimate and the Kappa agreement were similar to those in our study. The reasons for these false negative results are unclear, but one may speculate that if there are few IAV positive pigs within the litter, their viral RNA contribution to the sample may get diluted with the fluids of negative pigs in the litter, resulting in a false negative udder wipe.

Our results also showed a substantial agreement between oropharyngeal swab RT-qPCR results and nasal wipe qPCR results (Kappa = 0.8). This point estimate is higher than the one reported by Garrido-Mantilla et al. [19], who estimated the litter-level agreement between nasal wipes and oropharyngeal swabs at a Kappa value of 0.56. Nonetheless, the 0.56 kappa point estimate still lies within the 95% CI reported here. The estimated sensitivity of nasal wipes was 75.0% (95% CI 47.6–92.7%) when compared to oropharyngeal swabs, suggesting that about 25% of false-negative results can occur when using this sampling method to detect IAV at the litter level. Nasal wipes were proposed as a non-invasive method to assess the IAV status of pigs. Edwards et al. [30] showed that nasal wipes had a 92.9% sensitivity when compared to the RT-qPCR results of nasal swabs. Vendruscolo et al. [31] estimated the pig-level sensitivity of nasal wipes at 84% (95% posterior probability interval 70.0–95.0%) using a Bayesian Latent Class Model approach. Although our litter-level nasal wipe sensitivity estimate falls within the 95% posterior probability of the pig-level estimate reported by Vendruscolo et al. [31], it is possible that the aggregated sampling performed in our study may have introduced a dilution factor, increasing the probability of getting a false-negative result.

This study evaluated the sensitivity and specificity of udder wipes and nasal wipes compared to oropharyngeal samples originating from six pigs within a litter. In our design, it could have been possible that only negative pigs in an IAV-positive litter were sampled. For example, using the binomial distribution, if the within-litter prevalence was ~7% (1/14 positive pigs), then the possibility of sampling six negative pigs from that litter would have been 65% (i.e., litter-level sensitivity 35%). This litter-level false-negative percentage is reduced as the within-litter prevalence increases. For a within-litter prevalence of 43% (6/14 positive pigs), the possibility of sampling six negative pigs would have been 3.4% (litter-level sensitivity 96.6%). In our data, the median within-litter prevalence in RT-qPCR-positive litters was 66.7%. However, the real RT-qPCR status of negative litters and their within-litter prevalence is unknown. Since we used oropharyngeal swabs as the gold standard for litter classification, apparent false-positive results in the udder wipe litter classification may have occurred if the udder wipe was able to detect the virus in a litter when only negative pigs were selected for the oropharyngeal sampling in an IAV-positive litter. However, the occurrence of positive udder wipe results in litters classified as negative by oropharyngeal swabs was rare in our data (2/57 litters), suggesting that IAV was present at a relatively high within-litter prevalence at the time of sampling. Overall, we think that the sensitivity and specificity estimates reported here should be considered when assessing the IAV status of pigs or litters using nasal wipes or udder wipes. Furthermore, if the objective is to detect the virus at the room level, then these estimates can be used to choose the appropriate number of litters to sample, depending on the design prevalence selected to detect the virus within the room.

Since udder wipes are likely to represent the oral and nasal secretions of the pigs within a litter, it was expected that the Ct-value of positive udder wipes would be higher than the Ct-value observed in the individual pigs due to the dilution factor with fluids from negative pigs within a litter. The same was expected for nasal wipes, since an aggregated sample of three pigs was taken using a single wipe. However, our results showed a statistically non-significant increase of 0.56 and 0.48 Ct units for udder wipes and nasal wipes, respectively, with respect to the average observed in the individual oropharyngeal swabs. Similar results were observed by Moraes et al. [21] for the Ct-values of RT-qPCR-positive udder wipes and nasal wipes compared to Ct-values of RT-qPCR-positive family oral fluids (approximately 1–2 Ct-value unit increase). Garrido-Mantilla et al. [19] also observed a median increase of approximately two Ct-value units in udder wipe samples compared to pooled oropharyngeal swabs. However, Ct-values of nasal wipes had a median Ct-value slightly lower than that of pooled oropharyngeal swabs. Collectively, these findings suggest that the increase in Ct-values of udder wipes and aggregated nasal wipes is marginal compared to that of the individual pigs within the litter. Although we did not attempt to sequence the RNA of positive samples, Garrido-Mantilla et al. [19] showed that sequencing is possible, although the percentage of success is lower for group RT-qPCR-positive samples than for individual ones.

The positivity to IAV was observed to vary over the years in breeding herds A, B, and D. Herd C remained at a low prevalence in the two years sampled, whereas herd E was sampled only in 2023. IAV infection in pig populations is mainly determined by environmental, management, and animal factors. Chamba-Pardo et al. [32] observed that farms that introduced gilts positive for IAV to the breeding herd or those that did not have a vaccination protocol for sows have higher odds of weaning IAV-positive pigs than herds that introduced negative gilts or implemented a vaccination protocol for sows. Similarly, Diaz et al. [33] observed that new gilts and pigs at weaning had higher odds of testing IAV RT-qPCR positive than gilts introduced >4 weeks to the farm and that higher odds of positivity were observed during fall and winter compared to spring. These observations highlight the role of replacement gilts and pre-weaned pigs in maintaining the infection within the breeding herd.

Influenza in pigs has a seasonal pattern that has been observed to peak in late autumn–early winter and in spring in North America [34,35,36]. Although a similar pattern may be expected in commercial swine farms in Chile, IAV seasonality in the country has not been evaluated to date. Although farms were sampled in late autumn, marked changes in IAV RT-qPCR positivity were observed over the years, particularly in farms A and B, which decreased their positivity from 2022 to 2023. IAV positivity has been reported to vary substantially, even among farrowing rooms in the same farm on the same day of sampling [21]. Therefore, changes from one year to the next could be part of a random variation between farrowing rooms. Nonetheless, management changes or interventions implemented can have an impact on disease occurrence. In our study, farm A underwent a *Mycoplasma hyopneumoniae* elimination effort in 2022 that briefly consisted of mass exposure and farm closure for 10 months. This closure may have also contributed to the influenza RT-qPCR positivity reduction from 2022 to 2023 in that farm.

## 5. Conclusions

IAV prevalence in the Chilean swine breeding herds studied varied significantly over the years. Udder wipes and aggregated nasal wipes are group sampling methods that can be used to detect and monitor IAV in breeding herds. At the litter level, their RT-qPCR results correlate substantially to that observed in individual pigs using oropharyngeal swabs. Further research should evaluate the effect of litter level IAV prevalence on the performance of udder wipes. Nonetheless, although false negative results may arise at the litter level when using udder wipes or nasal swabs, the sensitivity and specificity values estimated here can be used to inform sample size calculations to determine the number of litters needed to be sampled to detect or quantify the presence of the virus at the farrowing room level or herd level.

## Figures and Tables

**Table 1:** Generalized estimating equations model results of the association between year of sampling or breeding herd of origin and RT-qPCR positivity for influenza A.

Variable	Levels	OR (95% CI)	*p*-Value
Year	2023	0.16 (0.03–0.84)	0.03
	2024	0.80 (0.22–2.94)	0.74
	2022	Reference	-
Breeding Herd	B	0.30 (0.07–1.25)	0.1
	C	0.02 (0.00–0.21)	0.001
	D	0.35 (0.09–1.43)	0.14
	E	2.50 (0.32–19.49)	0.38
	A	Reference	-

**Table 2:** Litter level influenza A status classification using udder wipes or oropharyngeal swabs as a sampling method.

	Oropharyngeal Swab (+)	Oropharyngeal Swab (−)
Udder wipe (+)	17	2
Udder wipe (−)	8	30
	Estimate % (95% CI)	
Sensitivity (%)	68.0 (46.5–85.1)	
Specificity (%)	93.8 (79.2–99.2)	
Positive predicted value (%)	89.5 (66.9–98.7)	
Negative predicted value (%)	78.9 (62.7–90.4)	
Positive predicted value of a negative test (%)	21.1 (9.6–37.3)	
Crude agreement (%)	82.5 (70.1–91.3)	
Kappa	0.63 (0.38–0.89)	

**Table 3:** Litter-level agreement between nasal wipe and oropharyngeal sampling.

	Oropharyngeal Swab (+)	Oropharyngeal Swab (−)
Nasal wipe (+)	12	0
Nasal wipe (−)	4	29
	Estimate % (95% CI)	
Sensitivity (%)	75.0 (47.6–92.7)	
Specificity (%)	100.0 (88.1–100.0)	
Positive predicted value (%)	100.0 (73.5–100.0)	
Negative predicted value (%)	87.9 (71.8–96.6)	
Positive predicted value of a negative test (%)	12.1 (3.40–28.2)	
Crude agreement (%)	91.1 (78.8–97.5)	
Kappa	0.80 (0.51–1.0)	

## Data Availability

Data are available on request due to restrictions because part of the data involves student thesis results and privacy concerns.

## Abbreviations

The following abbreviations are used in this manuscript:

IAV	Influenza A Virus
RT-qPCR	Quantitative Reverse Transcription PCRs
GEE	Generalized Estimating Equations
OR	Odds Ratio
